# Investigation of the mechanisms underlying the development and evolution of the mammalian cerebrum using gyrencephalic ferrets

**DOI:** 10.3389/fcell.2026.1803021

**Published:** 2026-04-01

**Authors:** Mayuko Yoshino, Angelina I. Mosiagina, Daisuke Sano, Reona Dei, Mao Yoshida, Hiroshi Kawasaki

**Affiliations:** 1 Department of Medical Neuroscience, Graduate School of Medical Sciences, Kanazawa University, Kanazawa, Ishikawa, Japan; 2 Sapiens Life Sciences, Evolution and Medicine Research Center, Kanazawa University, Kanazawa, Ishikawa, Japan

**Keywords:** cerebrum, cortical folds, development, evolution, ferret (*Mustela putorius furo)*, mammal

## Abstract

The mammalian cerebrum has changed notably during evolution, with increases in neurons and glial cells accompanied by its expansion and folding. Although these evolutionary changes are thought to be crucial for the acquisition of higher cognitive functions, the molecular and cellular mechanisms underlying the development and evolution of the mammalian cerebrum are still not fully understood. This is partly because of the difficulty in analyzing these mechanisms using mice only. To overcome this problem, genetic manipulation techniques for the cerebrum of gyrencephalic carnivore ferrets have been established. Gene knockout in the ferret cerebrum has also been achieved using the CRISPR/Cas9 system. In this review, we summarize recent research into the mechanisms underlying the development and evolution of the cerebrum using ferrets.

## 1 Introduction

**FIGURE 1:**
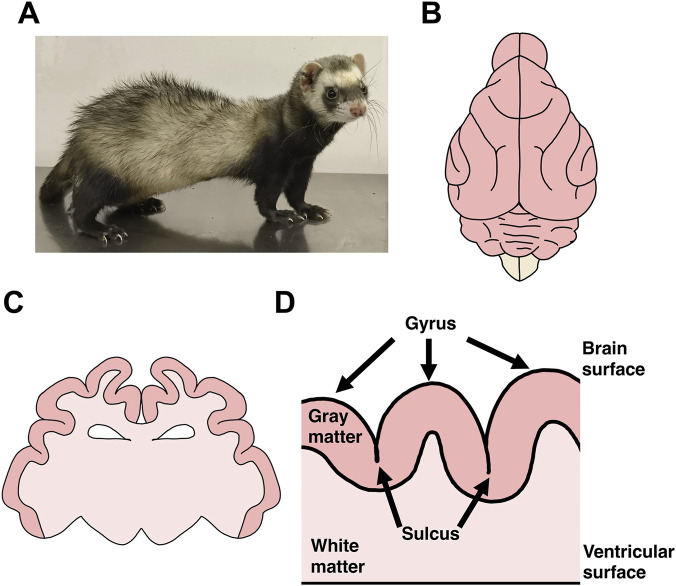
The ferret and its cerebrum structures. **(A)** A photograph of an adult ferret. **(B)** A schematic diagram of a dorsal view of the adult ferret brain. **(C)** A schematic diagram of a coronal section of the ferret brain. **(D)** A schematic diagram of a cross-section illustrating cortical folds.

The mammalian cerebrum has evolved through extensive transformation and diversification (Borrell and Gotz, 2014; Florio and Huttner, 2014; Gilardi and Kalebic, 2021; Mosti et al., 2025; Kawasaki, 2014; Kawasaki, 2017; Kriegstein et al., 2006; Lui et al., 2011; Molnar et al., 2006; Poluch and Juliano, 2015; Ra and kic, 1995; Rakic, 2009; Sun and Hevner, 2014; Zilles et al., 2013). During evolution, both neurons and glial cells increased in number, leading to the enlargement of the cerebrum. Furthermore, the cerebrum acquired complex structural features such as gyri and sulci, and its neural circuits became increasingly intricate. Although these changes are believed to be important for the emergence of higher cognitive functions, the mechanisms underlying the development and evolution of mammalian brains are still not fully understood. One of the challenges in this field has been the limitations of using mice, which are widely employed as standard model animals for genetic studies. The mouse brain is relatively small and lacks cortical folds, thereby limiting its utility for investigating the mechanisms of cortical folding and expansion. Consequently, alternative model animals are required to better understand cortical expansion and folding. Several research groups, including us, have used ferrets (*Mustela putorius furo*) for this purpose. Ferrets are medium-sized carnivores with a relatively large and well-developed cerebrum that contains cortical folds (Figures 1A–C) (Borrell et al., 2006; Fietz et al., 2010; Kawasaki et al., 2004; Neal et al., 2007; Noctor et al., 1999; Rowell et al., 2010; Smart and McSherry, 1986). In this review, we focus on recent studies into the mechanisms underlying cortical folding using ferrets. We also introduce recent findings on axon fiber layers in the developing cerebrum. Furthermore, we discuss the functional implications of evolutionary changes in the cerebrum.

## 2 Development of the mammalian cerebrum

**FIGURE 2:**
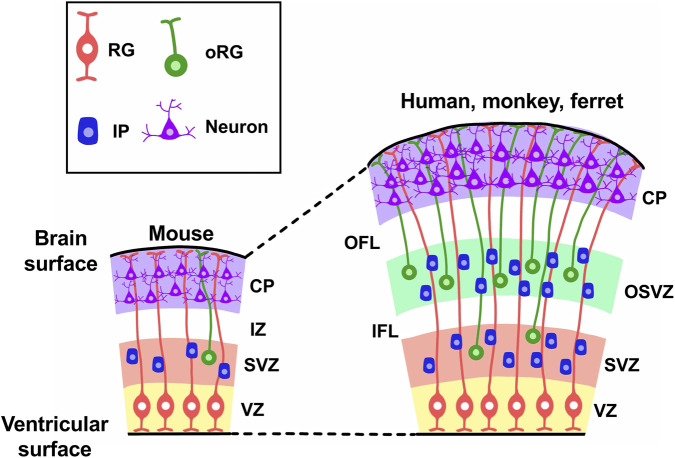
Neural progenitors in the developing cerebra of lissencephalic and gyrencephalic animals. The major types of neural progenitors present during cortical development, including radial glial (RG) cells, intermediate progenitor (IP) cells and outer radial glial (oRG) cells, are shown. In lissencephalic species such as mice, progenitors produce two germinal zones: the ventricular zone (VZ) and the subventricular zone (SVZ). In contrast, gyrencephalic animals such as humans, monkeys and ferrets possess an additional germinal zone, the outer subventricular zone (OSVZ). Abbreviations: CP, cortical plate; IZ, intermediate zone; OFL, outer fiber layer; IFL, inner fiber layer.

During development, neurons and glial cells in the cerebrum originate from neuroepithelial (NE) cells located around the lateral ventricles (Taverna et al., 2014). As development proceeds, NE cells transform into radial glial (RG) cells (also known as apical progenitor cells, ventricular RG (vRG) cells or apical RG (aRG) cells) within the ventricular zone (VZ) (Table 1). RG cells have bipolar radial processes that extend from the ventricular surface to the pial surface of the developing cerebrum, providing a scaffold for neuronal migration (Taverna et al., 2014). RG cells generate basal progenitors, which include intermediate progenitor (IP) cells and outer radial glial (oRG) cells (also referred to as outer subventricular zone (OSVZ) RG cells, basal RG (bRG) cells, intermediate RG cells or translocating RG cells) in the subventricular zone (SVZ) (Figure 2) (Table 1) (Taverna et al., 2014). Recent studies in the developing ferret cerebrum identified truncated RG (tRG) cells in the VZ and bipolar RG (bpRG) cells in the VZ and the SVZ (Bilgic et al., 2023; Pilz et al., 2013). tRG cells have also been reported in the developing human cerebrum (Nowakowski et al., 2016). These cells produce ependymal and astrogenic lineages and are characterized by truncated radial fibers terminating in the OSVZ (Bilgic et al., 2023). More recent work has shown that these cells also produce oligodendrocyte progenitors and olfactory bulb interneuron-destined intermediate progenitors (Yang et al., 2022).

**TABLE 1:** Neural progenitors in the germinal zones.

Cell types	Subtypes	Markers	Locations	Synonyms
oRG cells	​	Pax6+/Tbr2-	mainly OSVZ	OSVZ RG cells, bRG cells, intermediate RG cells, translocating RG cells
​	HOPX(+) oRG cells	Pax6+/Tbr2-/HOPX+	mainly OSVZ, mainly future gyri	​
​	HOPX(-) oRG cells	Pax6+/Tbr2-/HOPX-	mainly OSVZ	​
IP cells	​	Tbr2+	mainly ISVZ	​
bpRG cells	​	Pax6+	VZ, SVZ	​
tRG cells	​	Pax6+/Tbr2-	VZ	​
RG cells	​	Pax6+/Tbr2-	VZ	vRG cells, aRG cells,

In gyrencephalic species such as humans, monkeys and ferrets, the SVZ can be further divided into the OSVZ, which contains abundant oRG cells, and the inner SVZ (ISVZ) (Table 1) (Fietz et al., 2010; Nowakowski et al., 2016; Smart et al., 2002; Reillo and Borrell, 2012). In contrast, mice do not have a distinct OSVZ and possess only a small number of oRG cells in their developing cerebrum. Therefore, it has been proposed that an increase in oRG cells contributed to the expansion and folding of the cerebrum during evolution. Newly born cortical neurons derived from these precursor cells migrate toward the cortical plate using the radial processes of both RG cells and oRG cells. During cortical plate formation, late-born neurons migrate past early-born neurons, establishing the characteristic inside-out layering of the neocortex (Silva et al., 2019). As reported by numerous classic studies using mice, the generation of neurons in each cortical layer follows a chronological order, with lower-layer neurons being produced before upper-layer neurons (Noctor et al., 1997).

## 3 Structural and developmental features of cortical folds

The surface of the cerebrum in many mammals is characterized by cortical folds composed of gyri (ridges) and sulci (furrows), which are prominent structural features found in mammals with relatively large brains (Figure 1D). The acquisition of cortical folds during evolution substantially increased the surface area of the cerebrum and allowed more neurons to be accommodated within the skull. The increase in the cortical surface area is considered essential for the development of higher cognitive functions. Consistently, cortical malformation disorders, including polymicrogyria and lissencephaly, are frequently associated with severe intellectual disability (Ross and Walsh, 2001; Fernandez et al., 2016). Therefore, elucidating the molecular mechanisms underlying the development and evolution of cortical folds is not only a fascinating field of study but also critically important for gaining biological and medical insights into the brain.

Cortical folds are prominent in animal species with relatively large brains, including humans, monkeys and ferrets, whereas they are scarcely observed in species with smaller brains, such as rats and mice. The terms “gyrencephalic” and “lissencephalic” denote the presence and absence of these folds, respectively. The degree of cortical folding across mammals has been quantitatively assessed using the gyrification index (GI), where the GI of 1 indicates a lissencephalic brain, and higher GI values correspond to increasing levels of gyrification (Zilles et al., 1988). The GI differs across animal species, with reported values of 1.03 in mice, 1.63 in ferrets, 1.75 in monkeys and 2.56 in humans (Zilles et al., 2013). Investigating the mechanisms that determine whether the cerebrum becomes gyrencephalic or lissencephalic, as well as those that determine the extent of cortical folding, remains an important area of research.

Cortical folds exhibit unique structural features (Borrell, 2018). The curvature of the surface of gray matter and that of the boundary between gray matter and white matter leads to the alignment of all six cortical layers along the folds (Figure 1D). In contrast, the surface of white matter adjacent to the lateral ventricle remains relatively flat, resulting in thicker white matter beneath gyri compared to sulci. These distinct anatomical features should be carefully considered when analyzing cortical folding.

The formation of cortical folds occurs gradually during brain development and varies across gyrencephalic species. In humans and cynomolgus monkeys, cortical folds emerge prenatally, whereas they appear after birth in ferrets (Welker, 1990). The folding process begins with the formation of primary sulci, which are deep fissures located in defined positions. As the brain matures, secondary and tertiary sulci subsequently develop, giving rise to more complex folding patterns (Welker, 1990).

Mammalian species differ in the extent of these patterns. Animals with simple folding, such as ferrets, develop only primary sulci, whereas species with complex folding, such as humans, also form secondary and tertiary sulci. At the individual level, studies of identical twins have shown that the positions of primary sulci are highly conserved, while those of secondary and tertiary sulci vary (Lohmann et al., 1999). These findings suggest that the positions of primary sulci are largely determined by genetic factors, whereas those of secondary and tertiary sulci may be influenced by additional factors such as neuronal activity (Borrell, 2018).

## 4 Current working hypotheses on the mechanisms of cortical folding

Several hypotheses have been proposed to explain the genetic, cellular and mechanical mechanisms underlying cortical folding (Florio and Huttner, 2014; Gilardi and Kalebic, 2021; Mosti et al., 2025; Kawasaki, 2014; Kawasaki, 2017; Lui et al., 2011; Sun and Hevner, 2014; Borrell, 2018; Heuer and Toro, 2019). One hypothesis posits that intracranial pressure contributes to cortical folding. Because cortical folds are more prominent in animals with large cerebra, it was suggested that pressure within the skull may physically push the brain surface into folds (Welker, 1990). However, this appears unlikely because the mechanical effect of the skull on the brain seems to occur much later than cortical folding during development (Smart and McSherry, 1986). Another hypothesis is that axonal tension contributes to cortical folding. According to this model, tension generated by axon fibers connecting cortical areas pulls regions closer together, thereby creating folds between them (Van Essen, 1997). A third hypothesis is that the differential growth of cortical layers drives cortical folding (Kriegstein et al., 2006; Shinmyo et al., 2017; Richman et al., 1975). Preferential expansion of upper layers relative to lower layers may produce outward protrusions of the cortical surface, resulting in gyri. This hypothesis is supported by experiments using expandable gels, which reproduced fold-like structures when the outer layer expanded more than the inner core (Tallinen et al., 2016).

The discovery of oRG cells raised the possibility that these cells play a key role in cortical folding. In species with more folded brains, such as humans and monkeys, there tends to be a greater number of oRG cells compared to animal species with smoother brains (Figure 2) (Fietz et al., 2010; Dehay et al., 2015; Hansen et al., 2010; Reillo et al., 2011; Wang et al., 2011). Although the OSVZ is present in some species with limited folding, such as marmosets (Kelava et al., 2012), the strong correlation between the degree of folding and the number of oRG cells suggests that they play a key role in this process. Nevertheless, exceptions such as gyrencephalic rodents (e.g., agouti) indicate that additional mechanisms also contribute (Garcia-Moreno et al., 2012).

Finally, another hypothesis posits that the diversity of neural progenitors contributes to cortical folding. Gyrencephalic animal species often exhibit a greater variety of progenitor subtypes than lissencephalic species. This diversity may be linked to the process of cortical folding, and the growth activity and gene expression of these progenitors may also contribute to cortical folding (Reillo and Borrell, 2012; Reillo et al., 2011; Betizeau et al., 2013; de Juan Romero et al., 2015; Johnson et al., 2015; Matsumoto et al., 2017; Toda et al., 2016).

In summary, many hypotheses have been proposed, but testing them remained challenging because of the technical difficulty of manipulating gene expression in complex, gyrencephalic brains.

## 5 Ferrets as a model for research into the development and evolution of the cerebrum

Mice have been widely used to study the molecular mechanisms of cortical development, but there are limits to how much experiments using mice can contribute to our understanding of cortical folding because of their smooth brains. Nevertheless, mice remain popular in research because of the availability of various genetic tools, such as gene knockout and transgenic techniques. Previous studies in mice have reported several candidate genes that may be related to cortical folding (Florio et al., 2015; Ju et al., 2016; Liu et al., 2017). However, in order to clarify the roles of these genes, it is important to test their functions in animals that naturally develop cortical folds. Ferrets serve as a promising model for this purpose. Classified as medium-sized carnivorous mammals, ferrets are considered domesticated descendants of the European polecat (Figure 1A). They usually grow to about 50 cm in length, weigh 1–2 kg, and live on average for 6–10 years. They offer multiple advantages as a model for studying cortical development and folding.

First, ferrets have a relatively large and well-developed cerebrum with cortical folds, making them an excellent model for investigating folding mechanisms (Figures 1A–C). Second, ferrets have been widely used in electrophysiological and neuroanatomical studies, and as a result, a great deal of knowledge about their brain structure and function is available (Kawasaki et al., 2004; Borrell and Callaway, 2002; Callaway and Katz, 1993; Crowley and Katz, 2000; Cucchiaro and Guillery, 1984; Hahm et al., 1991; Huberman et al., 2003; Law et al., 1988; Meister et al., 1991; Mooney et al., 1993; Sur et al., 1988; Espinosa and Stryker, 2012; Katz and Crowley, 2002; White and Fitzpatrick, 2007). Studies of the ferret visual system, particularly the visual cortex and lateral geniculate nucleus, have revealed important insights into neural plasticity and critical periods (Espinosa and Stryker, 2012; Katz and Crowley, 2002; White and Fitzpatrick, 2007). The existing body of electrophysiological and anatomical data is useful for interpreting findings from genetic studies.

**FIGURE 3:**
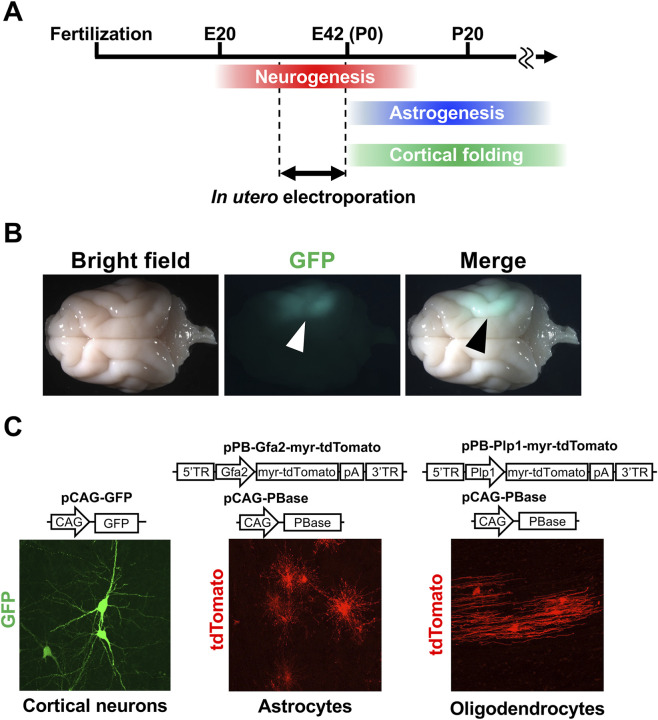
Genetic manipulation of the developing ferret cerebrum using *in utero* electroporation. **(A)** The time course of the development of the ferret cerebrum, highlighting the period suitable for *in utero* electroporation. Neurogenesis and astrogenesis progress during embryonic and early postnatal stages, respectively. Cortical folding occurs mainly after birth. **(B)** GFP expression in the ferret cerebrum induced by *in utero* electroporation. **(C)** Cell type–specific transgene expression. (left) neuronal expression. (middle) astrocyte-specific expression using the piggyBac system together with an astrocyte-specific promoter. (right) oligodendrocyte-specific expression using the piggyBac system together with an oligodendrocyte-specific promoter.

Third, ferrets are born at a relatively immature stage and continue to develop postnatally. Because neonatal brains are more accessible for experimental manipulation than embryonic brains, ferret pups are especially suitable for developmental studies. Importantly, whereas cortical folding is largely completed before birth in primates such as cynomolgus monkeys, it occurs after birth in ferrets (Figure 3), making them a practical model for directly examining the mechanisms of cortical folding.

Finally, in addition to neuroscience, ferrets are also widely used in other research fields, including studies of infectious diseases such as influenza and studies of vomiting mechanisms (Belser et al., 2011; Andrews and Hawthorn, 1987). Thanks to the knowledge accumulated about their care and breeding, they can be maintained with relative ease. However, despite these advantages, the lack of genetic manipulation techniques for ferrets had long posed a major limitation, preventing their full application in studies of the molecular mechanisms underlying brain development and evolution. Encouragingly, recent advances in this area are beginning to overcome these limitations, opening new opportunities to leverage the features of ferrets for investigating the development and evolution of the brain.

## 6 Genetic manipulation techniques for the ferret brain

Because ferrets provide unique advantages as a model system, the establishment of genetic techniques for the ferret brain had long been anticipated. Among the key foundational approaches in this field is genetic screening, which was greatly advanced by the development of a custom microarray using ferret cDNAs (Kawasaki et al., 2004). This provided a valuable platform for identifying genes with interesting expression patterns in the ferret brain. Using this approach, we successfully identified genes selectively expressed in magnocellular and parvocellular neurons, which are key components of the visual system in higher mammals (Kawasaki et al., 2004; Iwai et al., 2013; Sato et al., 2017). Genes with high expression in prospective gyral and sulcal regions of the developing ferret brain have also been reported (de Juan Romero et al., 2015). More recently, RNA-seq analyses revealed that gene expression patterns in ferret RG cells closely resemble those in human RG cells (Johnson et al., 2015). Furthermore, the availability of ferret genomic DNA and cDNA sequence data has greatly broadened the range of genetic tools available for ferret studies (Peng et al., 2014).

In addition to genetic screening techniques, the development of genetic manipulation techniques is essential for advancing studies of the molecular mechanisms underlying cortical development and evolution. Early attempts at gene introduction into the ferret cerebrum included postnatal electroporation and viral vector injections (Borrell, 2010; Nonaka-Kinoshita et al., 2013). Another key development was the establishment of *in utero* electroporation in ferrets, which provides a relatively simple and efficient method that can target most cortical neurons (Figure 3) (Kawasaki et al., 2012; Kawasaki et al., 2013). This technique enabled the transfection of multiple progenitor cell types, such as RG cells, IP cells and oRG cells (Kawasaki et al., 2012; Kawasaki et al., 2013). Importantly, because the procedure requires only about an hour per pregnant ferret, and transfected neonates can be obtained within several days, experimental progress is greatly accelerated. Another advantage of this technique is its flexibility. Multiple plasmids can be introduced simultaneously into the same brain, and various combinations of plasmids can be delivered into different embryos of a single pregnant ferret, allowing a wide range of experimental conditions (Kawasaki et al., 2012; Kawasaki et al., 2013). Using *in utero* electroporation in combination with the CRISPR/Cas9 genome editing system, both gene knockout and knock-in have been achieved in the ferret cerebrum (Shinmyo et al., 2017; Tsunekawa et al., 2016). Furthermore, combining *in utero* electroporation with the piggyBac system and cell type-specific promoters has enabled selective transgene expression in defined cell populations, such as astrocytes and oligodendrocytes (Figure 3C) (Hamabe-Horiike et al., 2021; Shinmyo et al., 2022).

Genomic modification provides another powerful technique for genetic manipulation in ferrets. Through genome editing–based knockout techniques, the functions of genes such as Aspm, Dcx and Disc1 in the ferret cerebrum have been elucidated (Johnson et al., 2018; Kou et al., 2015; Wang et al., 2024). Interestingly, the phenotypes of Aspm knockout ferrets were far more severe than those observed in mice, resembling clinical features in human patients, including pronounced microcephaly and abnormal positioning of RG cells (Johnson et al., 2018; Kou et al., 2015). In addition, transgenic ferrets have been generated by inserting foreign genes into the ROSA26 locus using the CRISPR/Cas9 system (Yu et al., 2019), expanding the genetic tools available for functional studies using ferrets. Beyond this, the successful generation of ferret induced pluripotent stem (iPS) cells has opened new avenues for *in vitro* modeling, including the development of brain organoids (Gao et al., 2020; Yosh et al., 2021). Collectively, these advances in ferret genetic modification, including knockout and knock-in approaches, transgenic animals and iPS cell technologies, have substantially broadened the scope of research possible in ferrets. As a result, ferrets are becoming an increasingly important experimental system for exploring the molecular mechanisms underlying brain development and evolution.

## 7 Research on the mechanisms underlying corticogenesis and cortical folding in ferrets

### 7.1 Roles of neural progenitors in cortical folding

Ferrets have recently emerged as an increasingly important model for investigating the molecular mechanisms that underlie brain development and evolution. Early pioneering studies demonstrated that neural progenitors are critical for cortical folding (see Section 2 for detailed information on neural progenitors). For example, it was shown that reducing the proliferation of these progenitors in the developing ferret brain suppresses cortical folding, whereas enhancing their proliferation promotes folding (Nonaka-Kinoshita et al., 2013; Haddad et al., 1979; Masuda et al., 2015). Interestingly, regional differences in progenitor distribution are evident even before folding begins. In the developing ferret cerebrum, future gyral regions contain larger numbers of neural progenitors than areas destined to become sulci (Reillo et al., 2011), and a similar pattern has been reported in monkeys (Smart et al., 2002). These findings suggest that progenitor enrichment in future gyral regions plays an important role in cortical folding. Nevertheless, experiments using lissencephalic mouse brains showed that increasing progenitor proliferation alone was not enough to produce cortical folds (Nonaka-Kinoshita et al., 2013). Therefore, in lissencephalic mouse brains, cortical folding appears to require not only an increase in neural progenitors but also additional developmental changes.

The developing cerebrum contains three main types of neural progenitors: RG cells (including vRG and tRG cells), IP cells and oRG cells (Table 1). Analyses of the developing ferret cerebrum have shown that IP cells and oRG cells are not evenly distributed; they are more abundant in future gyral regions compared to future sulcal regions (Reillo et al., 2011; Toda et al., 2016; Matsumoto et al., 2020). This distribution supports the hypothesis that more cortical neurons are produced by oRG and IP cells in future gyral regions, leading to the outward protrusion of gyri. A recent study identified two subpopulations of oRG cells: HOPX-positive and HOPX-negative (Table 1) (Matsumoto et al., 2020). HOPX-positive oRG cells are characterized by higher self-renewal capacity and are enriched in future gyral regions compared to HOPX-negative oRG cells. Interestingly, experiments that altered the number of HOPX-positive oRG cells showed corresponding changes in the degree of cortical folding. An increase in these cells led to more cortical folds, whereas a decrease produced the opposite effect (Matsumoto et al., 2020). These findings highlight the important role of HOPX-positive oRG cells in the process of cortical folding.

Recent RNA sequencing studies have uncovered the molecular diversity of RG cells in the developing ferret cerebrum. These studies found that this progenitor population can be subdivided into six major subclasses based on transcriptional features. Sulci are characterized by the presence of three subclasses (RGα_1_, RGβ_1_ and tRG), and gyri are characterized by the presence of two subclasses (RGα_2_ and RGβ_2_). One subclass (RGγ) is present in both gyral and sulcal regions (Del-Val et al., 2024). It was also shown that RGα subclasses are amplificative, whereas RGβ subclasses display a differentiative profile. Interestingly, the transcription factor Cux2 was predominantly found in RGα_1_ cells, and its overexpression in ferrets induced the formation of additional sulci (Singh et al., 2024). These results suggest that a transcriptomic protomap of neural progenitors, which is established during early development, predetermines the locations of future sulci and gyri (de Juan Romero et al., 2015; Del-Val et al., 2024).

### 7.2 Regulatory mechanisms of basal progenitor proliferation and differentiation

**FIGURE 4:**
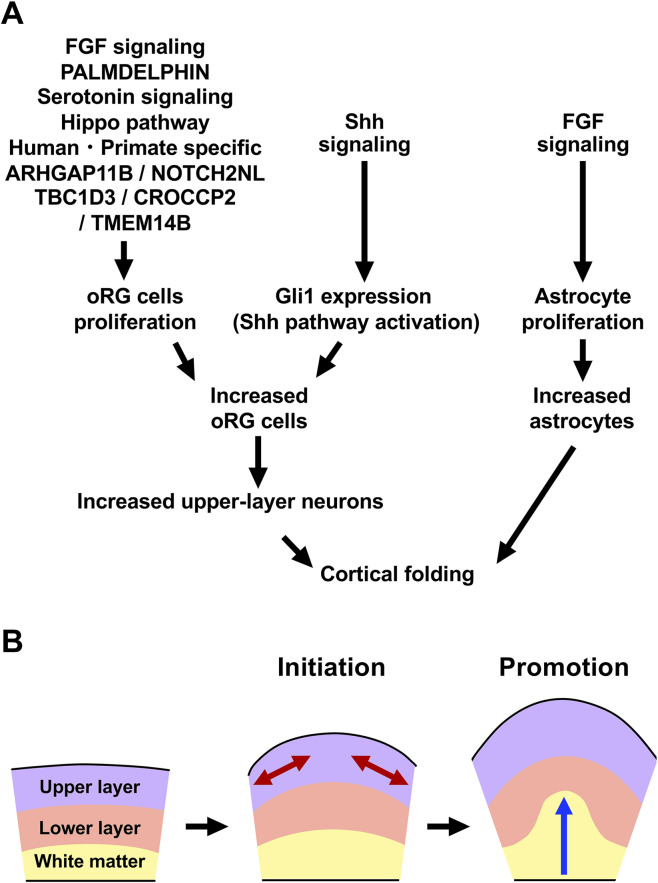
Our 2-step model describing the cellular mechanisms of cortical folding. **(A)** In gyrencephalic brains, the expansion of oRG cells—major contributors to upper-layer neuron production—initiates the formation of cortical folds. Then, astrogenesis subsequently supports further growth of cortical folds. **(B)** Cortical folding proceeds through two coordinated steps of tissue expansion: tangential enlargement of the cortical surface driven by a preferential increase in the upper layers (red arrows), followed by radial thickening of the cerebrum mediated by astrogenesis (blue arrow).

To elucidate the mechanisms that promote the proliferation and differentiation of basal progenitor cells in gyrencephalic brains, information about human diseases affecting cortical folding is useful, as it can reveals genes with critical roles in basal progenitor proliferation. One example is thanatophoric dysplasia, a congenital disorder that exhibits polymicrogyria. This disease is caused by a mutation in the fibroblast growth factor receptor 3 (FGFR3), which results in constant activation of FGF signaling, suggesting that FGF signaling is involved in neural progenitor proliferation and cortical fold formation (Shiang et al., 1994). This was confirmed in ferret models, where activating FGF signaling by *in utero* electroporation of FGF-expressing plasmids increased the proliferation of oRG cells in the developing ferret cerebrum (Figure 4A) (Masuda et al., 2015). Furthermore, inhibition of FGF signaling using a dominant-negative form of FGF receptor 3 reduced oRG proliferation (Matsumoto et al., 2017).

One study found that palmdelphin (PALMD), a member of the paralemmin protein family, is implicated in basal progenitor proliferation via integrin signaling (Figure 4A) (Kalebic et al., 2019). Introducing PALMD-CAAX into the developing ferret cerebrum led to a significant increase in basal progenitor proliferation (Kalebic et al., 2019). In the SVZ, PALMD-CAAX activated β1 integrin, which in turn activated the MAP kinase and PI3 kinase pathways. This suggests that the extracellular environment likely plays an important role in promoting the proliferation of basal progenitor cells.

Another study highlighted the role of serotonin signaling in basal progenitor proliferation (Figure 4A) (Xing et al., 2020). The serotonin receptor 2A (HTR2A) shows higher expression in gyrencephalic brains, such as those of ferrets and humans, compared with mouse brains (Xing et al., 2020). Overexpressing HTR2A in the mouse cerebrum increased the proliferation of basal progenitors, whereas HTR2A knockout in ferrets reduced it, suggesting that serotonin acting through HTR2A serves as an extrinsic signal promoting neural progenitor proliferation.

The Hippo pathway was also reported to play a role in regulating neural progenitor proliferation in the cerebrum (Figure 4A) (Kostic et al., 2019). The expression levels of YAP, a downstream effector of this pathway, was shown to be higher in the basal progenitors of ferrets and humans than in those of mice. Introducing constitutively active YAP into the mouse cerebrum promoted the proliferation of basal progenitors, while both pharmacological and genetic inhibition of YAP reduced the number of dividing basal progenitors in ferrets. These findings suggest that the increase in YAP levels, and presumably YAP activity, contributed to cerebral expansion during evolution (Kostic et al., 2019).

In addition, a recent study identified a role of the microRNA miR-3607 in regulating RG cell proliferation (Chinnappa et al., 2022). miR-3607 is expressed in the developing ferret cerebrum but not in the mouse cerebrum. In the embryonic mouse cerebrum, introducing miR-3607 enhanced Wnt signaling and stimulated RG cell proliferation, whereas loss of miR-3607 in ferrets reduced RG cell proliferation (Chinnappa et al., 2022). These results indicate that the number of RG cells can be regulated by microRNAs through species-specific modulation of signaling pathways.

### 7.3 Molecular and mechanistic insights into cortical folding

Recent studies have revealed how signaling pathways interact to regulate cortical folding, with a particular focus on fibroblast growth factor (FGF) and Sonic hedgehog (Shh) signaling. Both pathways are known to expand the population of oRG cells, which are crucial for cortical development (Matsumoto et al., 2017; Masuda et al., 2015; Matsumoto et al., 2020). In ferrets, activating FGF signaling by introducing an FGF-expressing plasmid into the developing cerebrum led to increased cortical folds, producing a phenotype similar to polymicrogyria (Masuda et al., 2015). Importantly, the induced cortical folds displayed normal laminar organization, with all 1-6 cortical layers present, and showed curvature on both the cortical surface and the gray–white matter boundary. In contrast, no curvature was observed on the ventricular surface. Conversely, inhibition of FGF signaling with dominant-negative FGF receptors inhibited cortical folding (Matsumoto et al., 2017). Similarly, activation of Shh signaling through Shh ligand introduction increased cortical folds, and inhibition of this pathway with HhipΔC22 suppressed cortical folding (Matsumoto et al., 2020). Moreover, inhibition of TMEM161B, a regulator of Shh signaling, also reduced cortical folding in ferrets (Akula et al., 2023). These findings suggest that FGF signaling and Shh signaling cooperate in regulating cortical folding (Figure 4A) (Matsumoto et al., 2017; Masuda et al., 2015; Matsumoto et al., 2020). A comparison between mice and ferrets showed that Shh protein levels are higher in the ferret cerebrum and found that Gli1 expression is higher in ferrets relative to mice (Matsumoto et al., 2020). These findings demonstrate that Shh signaling is more robustly activated in ferrets, and it is plausible that this stronger activity contributed to the expansion of oRG cells and the emergence of cortical folds during evolution.

An important question is how the increase of oRG cells translates into the morphological changes of cortical folding. Studies of brains with activated FGF signaling or Shh signaling showed that the upper cortical layers preferentially expanded vs. the lower layers (Matsumoto et al., 2017; Masuda et al., 2015; Matsumoto et al., 2020). These findings support the hypothesis that the ratio between upper and lower cortical regions is an important determinant of cortical folding. To investigate this further, one study used Cdk5 to selectively reduce the number of neurons in the cortical upper layers. A loss-of-function mutation in the Cdk5 gene has been identified in human lissencephaly patients, suggesting a role for Cdk5 in cortical folding (Magen et al., 2015). When Cdk5 was knocked out in pyramidal neurons of the developing ferret cerebrum using *in utero* electroporation combined with the CRISPR/Cas9 system, cortical folding was markedly suppressed (Shinmyo et al., 2017), indicating that Cdk5 plays an important role in this process. Cdk5 is essential for radial migration, and experiments introducing dominant-negative Cdk5 into layer 2/3 or layer 5/6 neurons revealed that blocking the migration of layer 2/3 neurons impaired cortical folding in ferrets, whereas blocking the migration of layer 5/6 neurons did not (Shinmyo et al., 2017). These findings indicate that cortical folding results from the preferential expansion of upper layers relative to lower layers (Figure 4B). To further strengthen this conclusion, it would be important to confirm that the number of cortical neurons is not affected by dominant-negative Cdk5. Furthermore, cortical neurons migrating in the developing ferret cerebrum show substantial tangential displacement rather than purely radial trajectories, suggesting a functional link between neuronal migration patterns and cortical folding (Gertz and Kriegstein, 2015).

Since cortical folding continues beyond the period of neurogenesis, it suggests that other factors are also involved (Figure 3A). Because astrocytes are generated after the completion of neurogenesis (Figure 3A), we hypothesized that astrogenesis contributes to cortical folding. Supporting this hypothesis, it has been shown that glial cell numbers increased as cortical folds developed, and the cerebrum expanded during evolution (Herculano-Houzel, 2014). To investigate the molecular mechanisms underlying the evolutionary increase of astrocytes, we compared gene expression patterns between mouse and ferret astrocytes and found that the expression level of FGF1 is higher in ferret astrocytes (Shinmyo et al., 2022). Ferret astrocytes also express the FGF receptors 2 and 3, suggesting that autocrine/paracrine FGF signaling promotes astrocyte proliferation through a positive feedback loop. Consistent with this, pharmacological inhibition of the FGF receptors using BGJ398 in primary ferret astrocyte cultures suppressed their proliferation (Shinmyo et al., 2022). To further examine the role of astrogenesis in cortical folding, we improved our *in utero* electroporation techniques. *In utero* electroporation typically results in transgene expression primarily in neurons (Kawasaki et al., 2012; Kawasaki et al., 2013), but when combined with the piggyBac system and cell type–specific promoters, it allowed selective gene manipulation in astrocytes and oligodendrocytes (Figure 3C) (Hamabe-Horiike et al., 2021; Shinmyo et al., 2022). When this system was used to reduce astrocytes, cortical folding was inhibited in ferrets (Shinmyo et al., 2022).

Based on these results, we proposed a 2-step model of cortical folding processes that consists of initiation and promotion (Figure 4B). During the initiation step, the preferential expansion of upper layers leads to the tangential expansion of the surface, resulting in the initial convolution of gyri. This step is mainly driven by neurogenesis. In the following promotion step, astrogenesis contributes to the vertical expansion of cortical folds (Figures 4A,B) (Shinmyo et al., 2022). Our 2-step model posits that two distinct steps cooperate to generate the full configuration of gyri and sulci.

### 7.4 Conserved and species-specific mechanisms of cortical folding

To better understand the evolutionary changes in the cerebrum, it is important to determine whether the mechanisms of cortical folding identified in ferrets are conserved in other gyrencephalic mammals, particularly primates. Evidence indicates that these processes are highly conserved between humans and ferrets. For example, FGF and Shh signaling pathways play important roles in cortical folding across gyrencephalic mammals, including ferrets and humans. Similarly, the microtubule-associated protein doublecortin (DCX) is essential for cortical folding in both species. This is evident in the fact that mutations in DCX are a known risk factor for lissencephaly in humans (Kato et al., 2003), and lissencephaly is also observed in DCX knockout ferrets (Wang et al., 2024). Comparative studies have further shown that gene expression patterns in prospective gyral and sulcal regions are remarkably similar between ferrets and humans (de Juan Romero et al., 2015). Moreover, miR-3607, which is crucial for the amplification of RG cells, is conserved across these species, indicating that the mechanisms of cortical folding are shared among gyrencephalic animals (Chinnappa et al., 2022).

Comparisons of the mechanisms underlying the development of the cerebrum among mice, ferrets and humans provide important insights into evolutionary changes. Shh activity is more strongly enhanced in the developing cerebrum of ferrets than in that of mice and mediates cortical folding (Matsumoto et al., 2020). The expression levels of YAP are higher in basal progenitors of ferrets and humans than in those of mice, and YAP promotes the proliferation of basal progenitors (Kostic et al., 2019). FGF1 is more highly expressed in ferret astrocytes than in mouse astrocytes and increases astrocyte numbers (Shinmyo et al., 2022). The microRNA miR-3607 is expressed in the developing ferret cerebrum but not in the mouse cerebrum, where it enhances RG cell proliferation (Chinnappa et al., 2022). These findings suggest that alterations in these mechanisms likely contributed to evolutionary changes in the cerebrum. Elucidating the complete molecular framework underlying the evolutionary changes of the cerebrum would be an important issue for future studies.

In contrast to the ferret cerebrum, the human cerebrum exhibits more highly developed structural features (Zilles et al., 2013; Hutsler et al., 2005). The human brain is not only significantly larger than the ferret brain but also more intricately folded, suggesting the existence of primate- and/or human-specific genes underlying cerebral development and evolution. Recent studies have identified such genes, including ARHGAP11B, NOTCH2NL, TBC1D3, CROCCP2 and TMEM14B, which are implicated in the amplification of neural progenitors (Florio et al., 2015; Ju et al., 2016; Liu et al., 2017; Arcila et al., 2014; Fiddes et al., 2018; Nowakowski et al., 2018; Van Heurck et al., 2023; Suzuki et al., 2018; Florio et al., 2018). Ectopic expression of these genes in the embryonic mouse cerebrum demonstrated that NOTCH2NL promotes the proliferation of apical/basal progenitor cells, whereas TBC1D3, CROCCP2 and TMEM14B are involved in the proliferation of basal progenitors and induce cortical folding. Introduction of ARHGAP11B into the ferret cerebrum resulted in further expansion of oRG cells and the cerebrum (Kalebic et al., 2018). These findings demonstrate how ferrets can be used to study the impact of primate- and human-specific genes on brain evolution.

In addition to ferrets, monkeys and humans, many other mammals also have cortical folds, and the degree of folding varies greatly among species (Zilles et al., 2013). Each species likely possesses its own distinct mechanisms that determine the extent of cortical folding. Currently, these mechanisms remain largely unknown, at least in part because genetic manipulation techniques are scarcely available in gyrencephalic animals other than ferrets. A key future direction will be to establish such techniques in other mammalian species and to compare the mechanisms of cortical folding across different animals.

## 8 Biological significance of cortical folds

**FIGURE 5:**
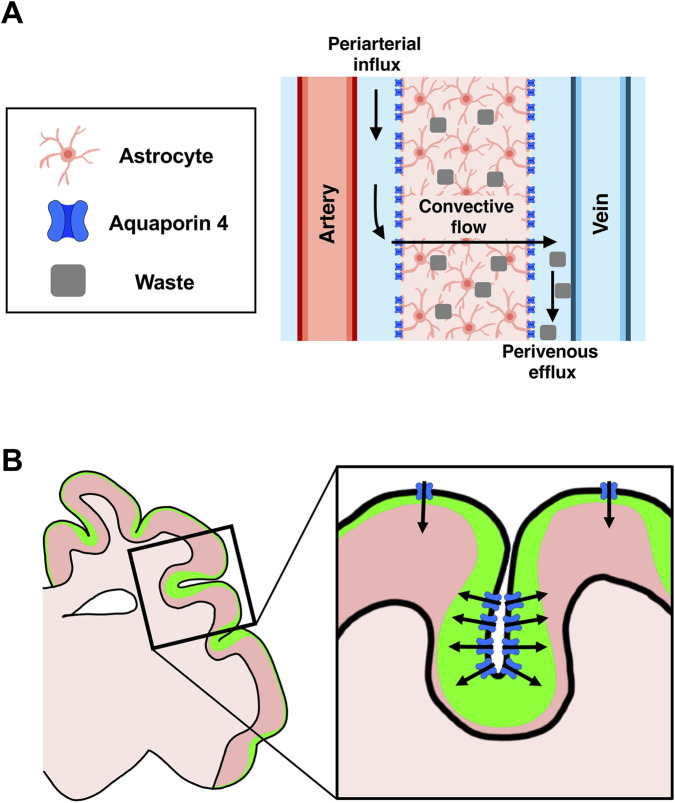
Biological role of cortical folds in glymphatic circulation. **(A)** CSF in the subarachnoid space enters the brain parenchyma through perivascular spaces (arrows), which are ensheathed by astrocytic endfeet. Aquaporin 4 expressed on astrocyte endfeet mediates CSF influx into the parenchyma. Within the parenchyma, CSF mixes with interstitial fluid, and the mixed fluid exits along venous perivascular spaces (arrows), contributing to the clearance of metabolic wastes such as amyloid β. **(B)** A schematic diagram of CSF influx patterns observed in the ferret cerebrum. CSF influx is stronger at sulci than at gyri, producing a sulcus-dominant CSF influx pattern (green). This sulcus-dominant CSF influx (arrows) is mediated by the accumulation of aquaporin 4-positive astrocytes at sulci.

The fact that the cerebra of many mammalian species are commonly covered with folds suggests the possibility that cortical folds themselves have some important roles besides just increasing the number of cortical neurons in the skull. A recent study investigated the involvement of cortical folds in the glymphatic system, which is a recently identified cerebrospinal fluid (CSF) circulation pathway (Figure 5A) (Mestre et al., 2020; Iliff et al., 2012). The CSF in the subarachnoid space enters the brain parenchyma through perivascular spaces, clearing metabolic waste including amyloid β from the brain parenchyma. Dysfunction of the glymphatic system has been linked to various brain diseases including Alzheimer’s disease (Harrison et al., 2020; Peng et al., 2016; Toh and Siow, 2021; G et al., 2014; Ferrara et al., 2022; Iliff et al., 2014). However, its evolutionary changes that occurred in the glymphatic system remain unclear because most studies have used mice (Mestre et al., 2020; Iliff et al., 2012).

It seemed plausible that as the cerebrum enlarged during mammalian evolution, CSF influx from the surface may have become inefficient in deeper regions of the cerebrum. We therefore hypothesized that enlarged brains possess yet undiscovered strategies to enhance CSF influx. To test this, we performed mathematical modeling and simulation of CSF influx patterns in the monkey and human cerebra (Kameya et al., 2024). Our simulation revealed that cortical folds enhance the efficiency of CSF influx. Furthermore, to investigate glymphatic circulation *in vivo*, we injected a CSF tracer into the CSF of the ferret brain. We found a novel CSF influx pattern; CSF influx was stronger at sulci than at gyri (Figure 5B) (Kameya et al., 2024). This sulcus-dominant CSF influx was mediated by the accumulation of aquaporin 4-positive astrocytes at sulci (Figure 5B). Importantly, the accumulation of aquaporin 4-positive astrocytes was also observed in the human cerebrum, suggesting that the human cerebrum also has sulcus-dominant CSF influx. These findings suggest that the enhancement of glymphatic circulation efficiency was a factor driving the evolution of cortical folds (Kameya et al., 2024).

## 9 Development and evolution of fiber layers in the cerebrum

**FIGURE 6:**
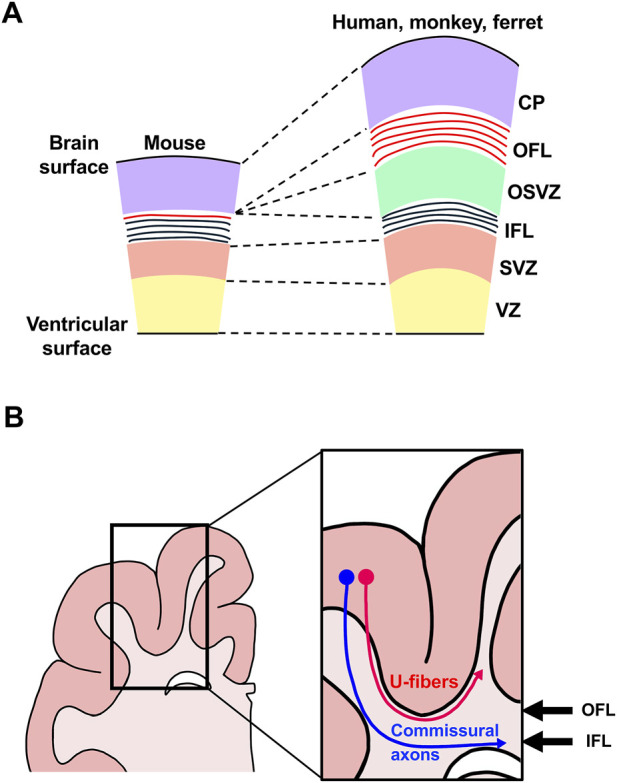
A proposed model for the evolutionary development of fiber layers in the cerebrum. **(A)** Schematic cross-sectional diagram of the developing cerebra. During mammalian evolution, axons forming the outer fiber layer (OFL, red) became progressively more abundant, giving rise to a prominent fiber band beneath the cortical plate. **(B)** Schematic cross-sectional diagram of the adult ferret cerebrum. Axons located in the OFL become subcortical U-fibers (red), whereas axons within the inner fiber layer (IFL) give rise to commissural fibers (blue) as well as subcortically projecting fibers. Abbreviations: CP, cortical plate; OSVZ, outer subventricular zone; SVZ, subventricular zone; VZ, ventricular zone.

In the developing human and monkey cerebrum, two distinct fiber layers emerge: the inner fiber layer (IFL) and the outer fiber layer (OFL) (Figure 6) (Molnar and Clowry, 2012); these two fiber layers have not been described in the mouse brain. The IFL separates the ISVZ and the OSVZ, whereas the OFL locates between the OSVZ and the cortical plates. In ferrets, when green fluorescent protein (GFP) was introduced into excitatory neurons by *in utero* electroporation, GFP-positive axons were observed in positions corresponding to the IFL and the OFL, closely resembling the organization seen in primate brains (Figure 6) (Saito et al., 2019), suggesting that, like humans and monkeys, ferrets also develop the IFL and the OFL during brain development.

To investigate neural circuits formed by axons within the IFL and the OFL, we traced the projection patterns of GFP-positive axons at different stages of ferret brain development. Our study showed that axons from the IFL mainly form commissural and subcortical projections, whereas axons from the OFL primarily develop into subcortical U-fibers (Figure 6) (Yoshino et al., 2020). U-fibers are short association fibers located just below gray matter and are especially abundant in humans and monkeys (Catani et al., 2012; Meynert, 1885; Nieuwenhuys et al., 1988; Ouyang et al., 2017; Schuz et al., 2002). These fibers are thought to mediate functional interactions between neighboring cortical regions and have been linked to various neurodevelopmental and psychiatric disorders. Research on U-fibers has been extensively conducted using MRI and histological studies in human and monkey brains, but genetic research on them remains limited. Hence, ferrets provide a valuable model for studying the development, function and pathophysiological roles of U-fibers (Yoshino et al., 2020; Yoshino et al., 2024).

Similar *in utero* electroporation experiments were also performed in mice. Although classical U-fibers have not been described in mice, a small number of GFP-labeled axons were observed extending into neighboring cortical regions of the mouse cerebrum (Saito et al., 2019). These findings suggest that U-fiber–like axons may also exist in mice, and that an increase in these axons likely contributed to the formation of the OFL (and as a result, U-fibers) as a thick axon bundle in the ferret and human cerebrum (Figure 6). Future studies using ferrets would be useful for elucidating the molecular mechanisms regulating U-fiber formation and for clarifying their physiological and pathological roles.

## 10 Future directions

Research on the mammalian cerebrum is critical for understanding its development, evolutionary history and higher cognitive functions. The introduction of advanced genetic technologies, such as *in utero* electroporation and CRISPR/Cas9-based genome editing, has greatly expanded the scope of studies in this field. While mice have served as the traditional foundation of genetic research, ferrets provide a valuable platform for *in vivo* analyses of mechanisms underlying the development, evolution and functions of the brain.

At the same time, it is important to recognize the limitations of ferrets. Incorporating human experimental models—particularly brain organoids derived from human iPS or embryonic stem (ES) cells—offers a promising strategy to overcome these limitations. In the future, integrative approaches that combine data from ferrets, human organoids, and other gyrencephalic mammals, such as non-human primates, would be important. Comparative studies are expected not only to clarify the mechanisms driving the development and evolution of the mammalian cerebrum but also to uncover the functional significance of cortical evolution.
